# Antimicrobial and Antioxidant Activities of Plants from Northeast of Mexico

**DOI:** 10.1093/ecam/nep127

**Published:** 2011-03-09

**Authors:** Ricardo Salazar-Aranda, Luis Alejandro Pérez-López, Joel López-Arroyo, Blanca Alicia Alanís-Garza, Noemí Waksman de Torres

**Affiliations:** Departamento de Química Analítica, Facultad de Medicina, U.A.N.L. Monterrey, P.O. Box 2316, Sucursal Tecnol579gico, 64841, N. L. México, Mexico

## Abstract

Traditional medicine has a key role in health care worldwide. Obtaining scientific information about the efficacy and safety of the plants from our region is one of the goals of our research group. In this report, 17 plants were selected and collected in different localities from northeast Mexico. The dried plants were separated into leaves, flowers, fruit, stems, roots and bark. Each part was extracted with methanol, and 39 crude extracts were prepared. The extracts were tested for their antimicrobial activity using three Gram-negative bacterial strains (*Pseudomonas aeruginosa*, *Klebsiella pneumoniae* and *Acinetobacter baumannii*), three Gram-positive bacterial strains (*Enterococcus faecalis* and two *Staphylococcus aureus* strains), and seven clinically isolated yeasts (*Candida albicans*, *C. krusei*, *C. tropicalis*, *C. parapsilosis* and *C. glabrata*); their antioxidant activity was tested using a DPPH free radical assay. No activity against Gram-negative bacteria was observed with any extract up to the maximum concentration tested, 1000 **μ**g ml^−1^. We report here for the first time activity of *Ceanothus coeruleus* against *S. aureus* (flowers, minimal inhibitory concentration (MIC) 125 **μ**g ml^−1^), *C. glabrata* (MICs 31.25 **μ**g ml^−1^) and *C. parapsilosis* (MICs between 31.25 and 125 **μ**g ml^−1^); *Chrysanctinia mexicana* against *C. glabrata* (MICs 31.25 **μ**g ml^−1^); *Colubrina greggii* against *E. faecalis* (MICs 250 **μ**g ml^−1^) and *Cordia boissieri* against *C. glabrata* (MIC 125 **μ**g ml^−1^). Furthermore, this is the first report about antioxidant activity of extracts from *Ceanothus coeruleus*, *Chrysanctinia mexicana*, *Colubrina greggii* and *Cyperus alternifolius*. Some correlation could exist between antioxidant activity and antiyeast activity against yeasts in the species *Ceanothus coeruleus*, *Schinus molle*, *Colubrina greggii* and *Cordia boissieri*.

## 1. Introduction

The plant kingdom has been the best source of remedies for curing a variety of disease and pain. This is why medicinal plants have played a key role in the worldwide maintenance of health. Traditional herbal medicine is intimately related to the Mexican popular culture; its use has origins based on ancestral knowledge. Natural products of higher plants are an important source of therapeutic agents; therefore, many research groups are currently screening the different biological activities of plants [1–3].

Mexico has an extensive variety of plants; it is the fourth richest country worldwide in this aspect. Some 25 000 species are registered, and it is thought that there are *∼*30 000 not yet described [4]. In particular, the northeast of Mexico, with its semiarid climate, has a great number and variety of wild plants grown under extreme climatic conditions [4]. It is well known that the constituents of medicinal herbs can vary greatly as a result of genetic factors, climate, soil quality and other external factors [5], particularly the semiarid climate from northeast of México causes the production of secondary metabolites different from those found in the same species grown under less extreme conditions.

In the course of our investigations, we have found that several plants from northwest Mexico possess interesting biological activities and could be a source of interesting new secondary metabolites [6–10].

The aim of this work was to investigate the antimicrobial and antioxidant properties of some wild plants grown in northeast Mexico used in the traditional medicine to treat infections or general ailments. In the current study, 39 extracts prepared from 17 plants belonging to different genera were submitted to antibacterial and antifungal assays using a microdilution method and to a test of antioxidant activity using the free radical scavenging activity of 1,1-diphenyl-2-picrylhydrazyl (DPPH) using both thin-layer chromatography (TLC) and spectrophotometric assay methods.

The activities have been selected because of their great medicinal relevance. It is well known that in past years, infection rates have increased and antibiotic resistance has become an increasing therapeutic problem [11, 12]. In addition a greater interest in the antioxidant activity of plant extracts exists because of free radicals (e.g., reactive oxygen species) that can be responsible for several diseases, for example, heart disease, stroke, arteriosclerosis and cancer, as well as the aging process [13].

## 2. Methods

### 2.1. Plant Materials

Wild plants were selected based upon their reported traditional use for the treatment of diseases including tuberculosis, fever, cough, hepatitis, rheumatism, skin diseases and respiratory or gastrointestinal infections. Collections were conducted between October 2000 and July 2002 from different regions in the states of Nuevo León and Coahuila (Table 1). Voucher specimens for each plant were deposited in the herbarium of the Facultad de Ciencias Biológicas, Universidad Autónoma de Nuevo León.

### 2.2. Extraction Procedure

The air-dried plants were separated into leaves, stems, roots, flowers, fruit and bark; each part was treated independently and immediately extracted. Powdered plant material (100 g) was extracted by direct maceration with methanol (3 × 600 ml) for 2 hours at room temperature. The extracts were filtered and evaporated to dryness under low pressure at 38°C. The different extracts were stored at 4°C until tested.

### 2.3. Test Organisms

The following were used as test organisms: *Candida albicans* (HU501), *C. albicans* (HU498), *C. albicans* (HU53), *C. krusei* (HU168), *C. tropicalis* (HU166), *C. parapsilosis* (HU96), *C. glabrata* (HU84), *Pseudomonas aeruginosa*, *Klebsiella pneumoniae*, *Acinetobacter baumannii* and *Enterococcus faecalis* (all provided by the Regional Center for Infection Diseases, Faculty of Medicine, UANL). In addition, two *Staphylococcus aureus* strains: a wild type (ATCC 12598) and an oxacillin-resistant strain (IMSS-NL/HE22:01) were also used.

### 2.4. Susceptibility Screening

Both bacteria and yeast strains were tested using a microdilution assay following the National Committee for Clinical Laboratory Standards (NCCLS) [24, 25], and minimal inhibitory concentration (MIC) values were determined. Bacterial strains were inoculated on Müeller–Hinton agar plates (Becton Dickinson) and were incubated at 37°C for 24 hours. Yeast strains were inoculated on Sabouraud agar plates (Becton Dickinson) and were incubated at 37°C for 24 hours. Four–five colonies were transferred from each solid culture to saline solution (3 ml) and then the solution was adjusted to 0.5 McFarland standard turbidity. The appropriate working suspension for each microorganism was prepared: a 1 : 50 dilution in Müeller–Hinton broth for bacteria and a 1 : 50 after 1 : 20 dilution in RPMI 1640 medium (Sigma-Aldrich) for yeasts, fresh medium recommended by NCCLS, was used in each case [24, 25]. The extracts were prepared at 4 mg ml^−1^ in 5% DMSO in the appropriate fresh liquid medium. Concentrations for each extract ranged from 1000 to 0.5 *μ*g ml^−1^. A 100 *μ*l volume of the extract sample was transferred to the first well of each row, and serial 2-fold dilutions were performed; the remaining 100 *μ*l was discarded. A 100 *μ*l volume of working suspension was added to each well. Seven samples and one antimicrobial drug control, cephalothin or fluconazole (500–0.25 or 62.5–0.25 *μ*g ml^−1^, resp.), were included in each plate. Three additional wells were used as growth controls where no drug was added, and culture medium was added to two wells. Plates were incubated at 37°C for 48 hours, and the growth was visually examined.

### 2.5. Determination of Antioxidant Activity

#### 2.5.1. Scavenging Activity of DPPH Free Radical by TLC

To measure antioxidant activity, DPPH free radical scavenging assay by TLC was used as previously reported [10]. Briefly, 10 *μ*l of each extract (1 mg ml^−1^ in ethanol) was applied to a chromatographic plate. Chromatography was conducted using ethyl acetate : acetic acid : formic acid : water (100 : 11 : 11 : 27) as eluent. The plate was developed using a DPPH solution (2 mg ml^−1^ in ethanol); 30 min later, the yellow spots from reduced DPPH were clearly observed against a purple background.

#### 2.5.2. Scavenging Activity of DPPH Free Radical by Spectrophotometry

Assay of DPPH scavenging activity by spectrophotometry was conducted according to Leu et al. [3], with some modifications. First, the extracts were redissolved in ethanol (1 mg ml^−1^), and different concentrations (200–0.234 *μ*g ml^−1^) of each extract were used. In a total volume of 1 mL, the assay mixture contained 500 *μ*l of the extract and 500 *μ*l of DPPH (125 *μ*M in ethanol). The assay mixture was shaken and allowed to stand at room temperature in darkness for 30 min. The absorbance was then measured at 517 nm in a DU 7500 spectrophotometer (Beckman Coulter). Quercetin was used as a positive control. The capacity to scavenge the DPPH radical was calculated as follows:

(1) $$\text{Radical scavenging activity }(\text{percent})=\frac{\left(A-B\right)}{A}\times 100,$$

where, *A* is the absorbance of the negative control (DPPH plus ethanol) and *B* is the absorbance of the sample (DPPH, ethanol plus sample). The correlation between each concentration and its percentage of scavenging was plotted, and the EC_50_ was calculated by interpolation. The activity was expressed as EC_50_ (the effective concentration of each extract that scavenges 50% of DPPH radicals).

## 3. Results

This article describes the antimicrobial and antioxidant activities of plants used in traditional medicine in northeast Mexico. A total of 39 extracts from 17 different plants species belonging to 11 families were tested. In Table 1, the botanical name, voucher specimen, part used, popular use and some chemotaxonomic criteria of the selected plant species are shown.

### 3.1. Antimicrobial Activity

**Figure 1:**
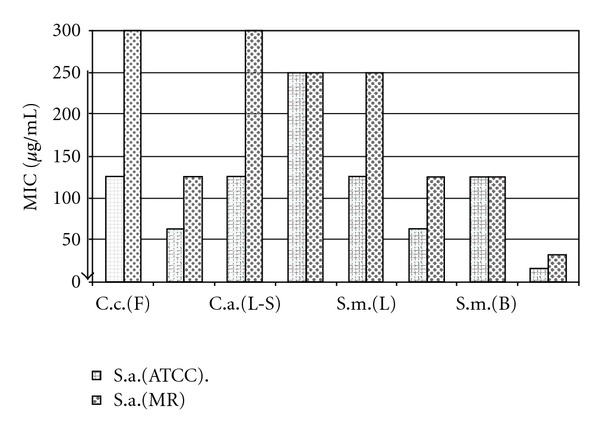
MIC of active extracts against S.a. *Staphylococcus aureus* strains: (ATCC) and an oxacillin-resistant strain (MR). L: leaves; F: flowers; R: root; L-S: leaves-steam; B: bark; C.c., *Ceanothus coeruleus*; C.m., *Chrysactinia mexicana*; C.g., *Colubrina greggii*; C.b., *Cordia boissieri*; C.a. *Cyperus alternifolius*; S.m., *Schinus molle*; Ctrl, control. The arrow shows better activity.

The results of the extracts displaying antimicrobial activity are shown in Table 2. No activity against Gram-negative bacteria (*P. aeruginosa*, *K. pneumoniae* and *A. baumannii*) was observed. The extracts from *Ceanothus coeruleus* (flowers), *C. boissieri* (flowers), *Cyperus alternifolius* (leaves, stem and root) and *Schinus molle* (leaves, flowers and bark) displayed the strongest activity against both sensitive and resistant *S. aureus* strains (Figure 1, MIC between 62.5 and 250 *μ*g ml^−1^), the flower extracts from *C. boissieri* and *Schinus molle* were the most active. Five extracts belonging to *Colubrine greggii* (leaves and flowers), *Cyperus alternifolius* (root) and *Schinus molle* (bark) displayed the strongest activity against *E. faecalis* (MIC between 125 and 250 *μ*g ml^−1^).

Most of the extracts tested exhibited some activity against *C. glabrata* (MIC ranged between 31.25 and 1000 *μ*g ml^−1^). All test microorganisms were more sensitive to the extracts obtained from *Ceanothus coeruleus* (leaves and root), *Colubrina greggii* (leaves, stem and roots) and *Schinus molle* (leaves and flowers) (Table 2).

### 3.2. Antioxidant Activity

To test their antioxidant activity, all the extracts were analyzed by a DPPH free radical assay using TLC. Only 12 extracts displayed a strong antioxidant activity on the chromatographic plate: *Ceanothus coeruleus* (leaves, flowers and root), *Chrysanctinia mexicana* (leaves), *Colubrina greggii* (leaves, flowers and root), *Cyperus alternifolius* (root), *Heliotropium angiospermum* (flowers and leaves-stem), *Phyla nodiflora* (leaves) and *Schinus molle* (bark). Smaller spots or spots with less intensity were displayed by three other extracts: *Cordia boissieri* (flowers), *Chrysanctinia mexicana* (flowers) and *Schinus molle* (flowers). The 15 active extracts were further tested for their scavenging activity using a DPPH spectrophotometric assay. The percentage reduction of DPPH radical exhibited by the different concentrations of each extract was calculated and subsequently its EC_50_ was determined (Table 3). Methanolic extracts from *Ceanothus coeruleus* (leaves and root), *Chrysanctinia mexicana* (flowers), *Colubrina greggii* (root), *Cyperus alternifolius* (root) and *Schinus molle* (bark), exhibited remarkable antioxidant activity with EC_50_s less than 10 *μ*g ml^−1^, while eight extracts displayed good antioxidant activities between 10.5 and 35.2 *μ*g ml^−1^. Quercetin was used only as a positive control for the antioxidant activity assay. The EC_50_ for quercetin was 3.0 *μ*g ml^−1^ (8.9 *μ*M), similar to the result reported by Torres et al. [26].

## 4. Discussion

The results of our investigation confirmed the rationale for the medicinal use of some of the studied plants. The plants were selected according to their popular use, and in few cases, chemotaxonomic criteria were used (Table 1).

The first remarkable aspect of the results obtained, was that no one of the extracts inhibited the growth of Gram-negative bacteria at the highest concentration tested. This lack of activity against Gram-negative bacteria is consistent with results discussed by other research groups [12]. In contrast, nine extracts obtained from five plants exhibited good or moderate activity against Gram-positive bacteria. These extracts displayed also excellent activity against at least one of the yeast under evaluation (Table 3).

Some of the results obtained are consistent with those reported by other authors such as the antibacterial activity of *Schinus molle* [8, 16, 27, 28] and the antioxidant activity of *H. angiospermum* [29].

In the present study, we demonstrated for the first time that the flowers from *Ceanothus coeruleus* are active against an antibiotic-sensitive *S. aureus* strain, as well as against *C. glabrata* and *C. parapsilosis*; the leaves and root extracts had significant activity against all tested yeasts; moreover, all the extracts from this plant exhibited a remarkable antioxidant activity (EC_50_ between 5.6 and 28 *μ*g ml^−1^). We could not find any previous report regarding antioxidant activity of this species.

Leaves and flowers extracts from *Chrysanctinia mexicana* displayed a very potent antioxidant activity (EC_50_ 10.5 and 8.3 *μ*g ml^−1^, resp.), as well as antifungal activity against *C. glabrata* (MIC 31.25 *μ*g ml^−1^). We could not find any report regarding activity against yeast or antioxidant activity from *Chrysanctinia mexicana* or other species belonging to the same species.

The leaves and flowers extracts from *Colubrina greggii* showed inhibitory activity against *E. faecalis* (MIC 250 *μ*g ml^−1^). The DPPH assay demonstrated highly potent antioxidant activity of leaves, flowers and roots from this plant (EC_50_ between 7.9 and 29 *μ*g ml^−1^). There were hitherto no reports regarding the antioxidant activity of any species from the *Colubrina* genus.

The root and leaf-stem extracts from *Cyperus alternifolius* showed a moderate activity against all yeasts tested, while the root extract produced a clear antioxidant activity (EC_50_ 9.3 *μ*g ml^−1^).

**Figure 2:**
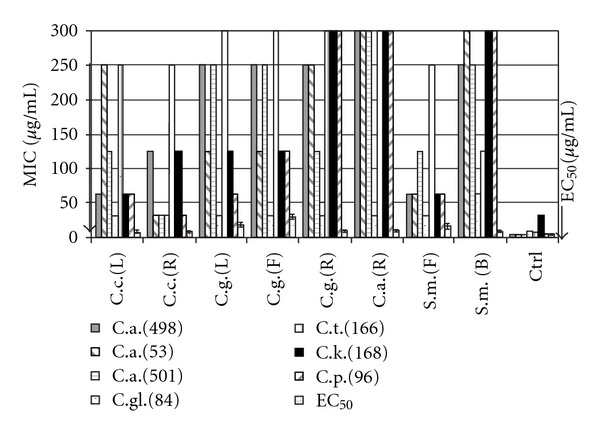
MIC of extracts active against yeast, and their antioxidant activity expressed as effective concentration (CE_50_). L: leaves; F: flowers; R: root; L-S: leaves-steam; B: bark; C.c., *Ceanothus coeruleus*; C.m., *Chrysactinia mexicana*; C.g., *Colubrina greggii*; C.b., *Cordia boissieri*; C.a. *Cyperus alternifolius*; S.m., *Schinus molle*; Ctrl, control; C.a., *Candida albicans*; C.gl., *Candida glabrata*; C.t., *Candida tropicalis*; C.p., *Candida parapsilosis*. The arrow shows better activity.

In summary, we conclude that most of the results of this study are in good agreement with the traditional uses of the investigated plants. All the extracts were active against *C. glabrata*. It appears that this isolate is sensitive to all the extracts used. Nine extracts showed activity against the rest of the yeasts tested; from these, eight extracts resulted with antioxidant activity (Figure 2). The root extract from *Ceanothus coeruleus*, displayed the greatest activity against yeast as well as the best antioxidant activity. Seven (46.6%) of the 15 extracts that showed antioxidant activity, displayed antibacterial activity as well, and nine (80%) resulted active against yeast. 

Belofsky et al. [30] demonstrated an increase in the antimicrobial activity of pure compounds when they are combined with antioxidants. Therefore, we consider that if both antimicrobial and antioxidant compounds exist in the extracts, they could interact and enhance the antimicrobial activity. The bioassay-guided fractionation of these extracts in order to isolate and identify the compounds responsible for each of these activities, followed by a study of their interaction, is highly desirable.

## Funding

Grant 103.5/06/0135 from PROMEP-México; U.A.N.L. (PAICYT) SA-1425-06.

## Figures and Tables

**Table 1:** List of plants screened.

Plant	Voucher	Family	Part	Popular use
*Ceanothus coeruleus* Lag.	024099	Rhamanceae	L, F, R	Fever, angina [14]
*Chrysactinia mexicana* Gray	024102	Compositae	L, F, R	Respiratory affections [15], against coldness of the spleen, tonic [4]
*Clematis drummondii* T&G.	024164	Ranunculaceae	L-S	Disinfectant, antibiotic, [4, 15], psoriasis, seborrhea, dermatitis, grains, freckles [4]
*Colubrina greggii* Wats.	024169	Rhamanceae	L, F, R	Fever, antibiotic [16], cough, tuberculosis [17]
*Cordia boissieri* A. DC.	024167	Borraginaceae	F	Cough, tuberculosis, respiratory affections [4, 15]
*Cyperus alternifolius* L.	024101	Cyperaceae	L-S, R	Aphrodisiac, stimulating [18]
*Eupatorium odoratum* L.	024100	Compositae	L, F, R	Chest complaints, pulmonary affections [15]
*Heliotropium angiospermum* Murr	024172	Borraginaceae	L, F-S	Cough, anticatharral [17]
*Leucophyllum frutescens* (Berl.) I.M.Johnst.	024165	Escrophulariaceae	F	Tuberculosis, bronchitis, diarrhea, damage of the liver, jaundice, fever [4].
*Phyla nodiflora* (L.) Greene.	024168	Verbenaceae	L, R	Antibacterial, diuretic, emmenagogue, parasitic [19]
*Porlieria angustifolia*	024173	Zigophylaceae	L, R	Tuberculosis, cough, cold, rheumatism, fall of hair, venereal diseases [4]
*Rivinia humilis* L.	024170	Phytolacaceae	L, F-Fr, R	Dermatological [20], against *P. aeruginosa*, *K. pneumoniae* and *S. aureus* [21]
*Salvia chia* Fernald	024103	Labiatae	L, F, R	Antioxidant, pharyngitis [18, 22]
*Salvia coccinea* Murr	024096	Labiatae	L, R	Tonsillitis, antimicrobial [18, 22]
*Salvia reflexa* Hornem	024095	Labiatae	L, F	Antioxidant, pharyngitis, tonsillitis, antimicrobial [18, 22]
*Schinus molle* L.	024166	Anacardiaceae	L, F, B	Pus, helps to eyes, teeth and mouth [4, 23]
*Scutellaria ellptica* Muhl	024104	Labiatae	L-S, F, R	Cough, expectorant, fever, cold [8]

L: leaves; F: flowers; B: bark; R: root; S: stem; Fr: fruit.

**Table 2:** Antimicrobial activity of extracts against bacterial strains and yeast isolates tested using on microdilution assay.

Plant	Part	*S. a*.	*S. a*.	*E. f*.	*C. a*.	*C. a*.	*C. a*.	*C. g*.	*C. t*.	*C. k*.	*C. p*.
		*ATCC*	Rsist.		498	53	501	84	166	168	96
*Ceanothus coeruleus*	L	—	1000	–—	62.5	250	125	31.25	250	62.5	62.5
	F	125	—	—	1000	1000	—	31.25	1000	1000	125
	R	—	1000	—	125	31.25	31.25	31.25	250	125	31.25
*Chrysactinia mexicana*	L	1000	—	—	—	—	—	31.25	—	—	–
	F	1000	—	—	—	—	—	31.25	1000	—	–
	R	1000	—	—	—	—	—	31.25	—	—	–
*Colubrina greggii*.	L	1000	—	250	250	125	250	31.25	500	125	62.5
	F	1000	—	250	250	125	250	31.25	500	125	125
	R	—	—	—	250	250	125	31.25	500	1000	500
*Cordia boissieri*	F	62.5	125	—	—	—	—	125	—	—	–
*Cyperus alternifolius*	L-S	125	—	1000	500	1000	500	31.25	1000	—	1000
	R	250	250	125	500	1000	500	31.25	500	500	1000
*Schinus molle*	L	125	250	1000	125	125	125	31.25	500	500	250
	F	62.5	125	1000	62.5	62.5	125	31.25	250	62.5	62.5
	B	125	125	125	250	500	250	62.5	125	1000	1000
Cephalotin		15.6	31.25	31.25							
Fluconazole					4	4	4	8	0.5	31.25	1

MIC values in *μ*g ml^−1^; MIC > 1000 *μ*g ml^−1^; L: leaves; F: flowers; B: bark; R: root; S: stem; *S. a. Staphylococcus aureus*; *E. f. Enterococcus, faecalis*; *C. a. Candida albicans*; *C. g. Candida glabrata*; *C. t. Candida tropicalis*; *C. k. Candida krusei*; *C. p. Candida parapsilosis*.

**Table 3:** Effects of extracts and positive control on the *in vitro* free radical (DPPH) scavenging activity (*μ*g ml^−1^).

Plant	Part	CE_50_ (*μ*g ml^−1^)
*Ceanothus coeruleus*	L	8.3 ± 1.0
	F	28.0 ± 3.0
	R	5.6 ± 2.9
*Chrysactinia mexicana*	L	10.5 ± 1.1
	F	8.3 ± 1.0
*Colubrina greggii*	L	18.3 ± 3.2
	F	29.0 ± 3.7
	R	7.9 ± 1.7
*Cordia boissieri*	F	105.1 ± 2.3
*Cyperus alternifolius*	R	9.3 ± 0.6
*Heliotropium angiospermum*	F	35.2 ± 2.1
	L-S	29.4 ± 1.4
*Phyla nodiflora*	L	20.8 ± 2.21
*Schinus molle*	F	15.2 ± 4.0
	B	8.6 ± 1.6
Quercetin (positive control)		3.0 ± 1.3

L: leaves; F: flowers; B: bark; R: root; S: stem.
